# Individual Prognosis of Symptom Burden and Functioning in Chronic Diseases: A Generic Method Based on Patient-Reported Outcome (PRO) Measures

**DOI:** 10.2196/jmir.8111

**Published:** 2017-08-01

**Authors:** Niels Henrik Ingvar Hjollund

**Affiliations:** ^1^ WestChronic Occupational Medicine, University Research Clinic Aarhus University Herning Denmark; ^2^ Department of Clinical Epidemiology Aarhus University Hospital Aarhus Denmark

**Keywords:** chronic disease, cohort studies, depression, longitudinal studies, patient-reported outcome measures, prognosis, recovery of function, repeated measurements, stroke, surveys and questionnaires, symptom assessment

## Abstract

**Background:**

Information to the patient about the long-term prognosis of symptom burden and functioning is an integrated part of clinical practice, but relies mostly on the clinician’s personal experience. Relevant prognostic models based on patient-reported outcome (PRO) data with repeated measurements are rarely available.

**Objective:**

The aim was to describe a generic method for individual long-term prognosis of symptom burden and functioning that implied few statistical presumptions, to evaluate an implementation for prognosis of depressive symptoms in stroke patients and to provide open access to a Web-based prototype of this implementation for individual use.

**Methods:**

The method used to describe individual prognosis of a PRO outcome was based on the selection of a specific subcohort of patients who have the same score as the patient in question at the same time (eg, after diagnosis or treatment start), plus or minus one unit of minimal clinically important difference. This subcohort’s experienced courses were then used to provide quantitative measures of prognosis over time. A cohort of 1404 stroke patients provided data for a simulation study and a prototype for individual use. Members of the cohort answered questionnaires every 6 months for 3.5 years. Depressive symptoms were assessed by the Hospital Anxiety and Depression Scale (HADS) and a single item from the SF-12 (MH4) health survey. Four approaches were compared in a simulation study in which the prognosis for each member of the cohort was individually assessed.

**Results:**

The mean standard deviations were 40% to 70% higher in simulated scores. Mean errors were close to zero, and mean absolute errors were between 0.46 and 0.66 SD in the four approaches. An approach in which missing HADS scores were estimated from the single-item SF-12 MH4 performed marginally better than methods restricted to questionnaires with a genuine HADS score, which indicates that data collected with shorter questionnaires (eg, in clinical practice) may be used together with longer versions with the full scale, given that the design includes at least two simultaneous measurements of the full scale and the surrogate measure.

**Conclusions:**

This is the first description and implementation of a nonparametric method for individual PRO-based prognosis. Given that relevant PRO data have been collected longitudinally, the method may be applied to other patient groups and to any outcome related to symptom burden and functioning. This initial implementation has been deliberately made simple, and further elaborations as well as the usability and clinical validity of the method will be scrutinized in clinical practice. An implementation of the prototype is available online at www.prognosis.dk.

## Introduction

Prognosis may be defined as foreseeing, predicting, or estimating future outcome based on the patient’s clinical profile [1]. The importance of individual prognosis was emphasized already by Hippocrates [2] at a time when effective medicinal treatments were rarely at hand and the principal role of the physician was to evaluate an illness and predict its likely progression based on information collected in detailed case histories. Prognostic evaluations are still an extremely important and integrated part of clinical practice, although in modern medical research, prognostic research has received less attention compared with therapeutic and etiological research [1,3]. As an example, a prognostic model may be developed to predict the short-term outcome after intracerebral hemorrhage. Early survival is known to be strongly dependent on the Glasgow Coma Scale score on admission [4]. Other factors that are known to predict outcome are the size of the hemorrhage and presence of intraventricular hemorrhage [5]. The prediction of outcome in patients with intracerebral hemorrhage can be used in the emergency department for decision support to differentiate between patients who might benefit from intensive care and those who have such poor prognosis that they will not benefit from intensive care [5]. However, another important use of prognostic knowledge is to inform the stroke patient and relatives [5].

### Prognosis Based on Patient-Reported Outcomes

In medicine, prognosis commonly relates to the occurrence of specific binary events such as death, relapse of disease, readmission, or specific complications [3,6], and scientific guidelines for prognosis research also focus on the prediction of binary events [7,8]. However, many outcomes are of continuous nature and highly relevant outcomes such as symptom burden and functioning cannot be assessed from clinical data only, but by application of patient-reported outcome (PRO) measures [9]. Information on the prognosis of disease-specific and general symptoms as well as functioning are often crucial for patients, and sometimes needed in order to make important decisions such as retirement or return to work, or whether to move to another house or flat. Social authorities and pension boards also need information on the prognosis of symptoms and functioning to make decisions that substantially affect the patient’s future life circumstances. Therefore, requests for prognosis regarding such outcomes are frequently made and every physician is familiar with answering such requests, formally or informally. However, such answers most often rely entirely on the individual physician’s personal clinical experience, attitudes, and beliefs, and only rarely on relevant quantifiable prognostic data, still less on biostatistical models [10]. This is somewhat surprising considering the demands for evidence-basedness. Depression is common after stroke, affecting approximately one-third of stroke survivors at any one time after stroke, compared with 5% to 13% of adults without stroke, with a cumulative incidence of 55% [11]. Therefore, the prognosis of depressive symptoms after stroke was selected for this study.

### Methodological Shortcomings

This lack of useful methods for individual PRO-based prognosis may be attributed to two reasons: lack of relevant data and the inadequacy of traditional statistical methods for constructing prognostic models. The very purpose of a statistical model is to reduce the original data to a few parameters (eg, estimated regression coefficients). However, such models may explain only a small percentage of the variation over time and sometimes none at all. In the latter case, we label such a study as “negative,” meaning that the group mean’s association with time was not statistically significant. This way of thinking reflects our focus on group means, not variations. Characteristically, we label unexplained variation as “error”—a noise that we failed to eliminate. However, individual variation is a natural phenomenon in all aspects of life and we should describe it, not eliminate it. From the patient’s perspective, it is not sufficient to be informed about group means. He or she would more likely prefer to be informed as much as possible about what courses similar patients have experienced. Furthermore, the traditional approach has a number of methodological limitations. A statistical model implies a number of presumptions about distribution, which may not be fulfilled. In addition, model building is complicated, especially if there are more than two measurements per patient [6]. However, given that relevant PRO data have been systematically collected, another possibility exists which addresses these obstacles. This paper describes a generic method that utilizes the cohort’s experience, implies few statistical presumptions, and is easy to extend to other outcomes and patient groups if relevant PRO data are available.

### Objective

The aim of this paper was to describe a simple and generic nonparametric, data-based method for on-the-fly individual prognosis that focuses on group means and variation to evaluate an implementation for prognosis of depressive symptoms in stroke patients, and to provide open access to a prototype for individual use.

## Methods

The principle used was for a given patient (named “the recipient”; ie, the patient for whom the prognosis is requested) to select the subcohort of patients (named “donors”) who have a score matching the patient’s score for the variable in question at the same number of days (named the “index day”) after the primary event (eg, diagnosis, treatment start). The criterion for donor match was the recipient patient’s value at the index date plus or minus a value corresponding to the minimal clinically important difference (MCID). The MCID may be either anchor based or distribution based [12]. In order to preserve the generic approach, the latter approach was preferred and calculated as one-half the standard deviation of the distribution of scores in cohort members [12,13]. The match criterion was applied for the cohort member’s last measurement before the index day. The subsequent trajectories for each member of the subcohort (donors) were simultaneously displayed and quantitatively described with summary statistics.

### Type of Questionnaires

Depressive symptoms were measured using the Hospital Anxiety and Depression Scale (HADS) [14]. This scale was developed to identify states of anxiety and depression among hospital outpatients; to avoid potential confounding by somatic illness, the construct excludes somatic symptoms such as insomnia and loss of energy [14]. The HADS consists of two subscales: an anxiety scale (HADS-A) and a depression scale (HADS-D). Each subscale includes seven items rated on a four-point rating scale (range 0-3), higher scores indicating more symptoms. Symptoms of anxiety and depression are assessed by summing the points within each subscale (0-21). Only the depression scale was used in this study. Cut-off values were used as proposed by Singer et al [15]. A maximum of two missing values were allowed by using the individual subscale means as proposed by Bell et al [16]. Two types of questionnaires were used: full-length and brief. Full-length questionnaires included the HADS [14], Multidimensional Fatigue Inventory (MFI-20) [17], WHO-5 Well-Being Index [18], and the 12-item Short Form Medical Outcomes Study (MOS SF-12) [19]. The brief questionnaire included MOS SF-12 as the only scale.

### Estimation of HADS-D Score in Brief Questionnaires

The HADS-D scores for brief questionnaires and for full-length questionnaires with missing HADS-D scores were estimated based on the MOS SF-12 MH4 item: “How much of the time during the past 4 weeks did you feel downhearted and depressed?” with the answer categories “all of the time,” “most of the time,” “some of the time,” “a little of the time,” and “none of the time.” In one approach, the regression estimates for the model HADS-D=β_0_+ β_1_ * SF-12 MH4 was calculated based on all patients (common regression). In another approach, regression estimates were calculated separately for each patient with at least three concurrent measurements of HADS-D and MOS SF-12 MH4 (individual regression). Finally, in the last approach, genuine HADS-D scores were used when present, supplemented with scores based on individual regression where genuine scores were missing.

### Simulation

Internal simulation was used to compare the four approaches: (1) genuine HADS-D score, (2) common regression-based scores, (3) local regression-based scores, and (4) a combined approach, where genuine scores were used, when available, otherwise local regression-based scores were inserted. Each patient was successively selected as a recipient with an index day defined as the date of the first measurement with a genuine HADS-D score. This day was treated as index date for the simulation of prognosis for that recipient patient. Donors were selected as described previously after deleting the actual recipient from the donor cohort. For each such simulation and for each time category, the numbers of measurements for the recipient as well as the donors were recorded as well as the differences between recipients’ actual scores and the mean value of donor scores. The intraindividual variation in donor and recipients was also recorded. In the first three approaches, only questionnaires with a valid HADS-D score were included, whereas the present SF-12 MH4 scores from the same patients were allowed in the last combined approach. Recipient patients were excluded if the subcohort of donor patients contained less than four members. For comparability reasons, the common regression-based and local regression-based simulation approaches were restricted to patients and questionnaires included in the genuine HADS-D approach.

### Evaluation

The performance of the four approaches was evaluated by comparing the values of means, standard deviations, intraindividual variation, mean error, and mean absolute error (MAE) between recipients and donors and across simulated approaches. MAE is a measure of forecast error in time series analysis [20], which unlike the mean square error, weights deviations proportionally. The Wall statistic was used to evaluate differences in mean scores between the four approaches and the donor population. The R version 3.2.5 package [21] was used for analyses, and the prototype runs on the server version of R-studio version 1.0.136 [22].

### Patient Population and Data Collection

**Figure 1:**
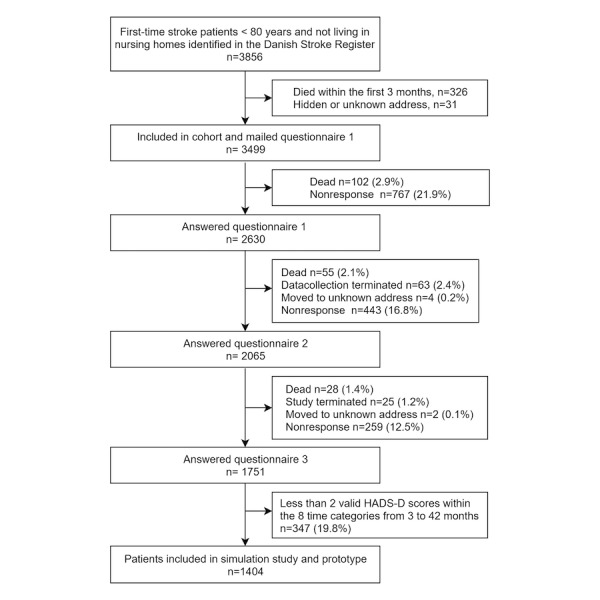
Flowchart of inclusion of stroke patients used in a data-based method for individual prognosis of depression.

The source population consisted of all patients with first-time stroke admitted to any hospital in the Central Denmark Region between October 1, 2008 and December 31, 2011. Patients were identified from the Danish Stroke Register, a nationwide initiative to monitor and improve the quality of care. Participation is mandatory for all Danish hospital departments treating patients for acute stroke. The register has been found to be valid regarding patient registration [23]. Patients younger than 80 years, alive 90 days after stroke, and living in their own homes before the stroke were included and invited to participate. Patients were identified in the register by their unique civil registration number. Information on gender and age at the time of stroke was obtained from the civil registration number. Information on comorbidity was retrieved from the Region Central Denmark patient registry. Information on address and vital status was collected from the Civil Registration System before approaching each patient [24]. Detailed information on the original cohort can be found elsewhere [25]. Data were collected by the WestChronic PRO system, which allows automated data collection with use of Web- and paper-based questionnaires. The system has in previous studies achieved response rates up to 93% for initial questionnaires and 98% to 99% for subsequent questionnaires [10]. The patients answered the initial questionnaire 3 months after the stroke, subsequently followed with repetitive questionnaires every 6 months until at least 3.5 years had elapsed. The first questionnaire was paper-based, but patients were encouraged to answer subsequent questionnaires online. Nonrespondents at a given time were mailed the brief version at the next scheduled date. The HADS scale was not included in the brief questionnaire, which, apart from the first 4 months of the study, was used as the initial questionnaire and when questionnaires were sent to patients who did not respond to the latest questionnaire. For each questionnaire, a time variable was assigned counting the number of days from the date of the stroke to the date the questionnaire data were received. For analyses and tabulation, the time was categorized in one of eight time categories with the best fit (3, 6, 12, 18, 24, 30, 36, and 42 months) by a method described elsewhere [26]. In all, 3856 patients fulfilled the inclusion criteria and 3499 were mailed a questionnaire 3 months after their stroke (Figure 1).

At least three questionnaires with at least two valid HADS-D scores were required for inclusion in the simulation study. A total of 1751 patients answered within at least three time categories, and 1404 patients had at least two valid HADS-D scores and were included in the simulation study (Figure 1). In some cases, a patient had more than one questionnaire in a time category and the second measurement was omitted in the simulation study.

The study was approved by The Danish Data Protection Agency (J.no. 2007-41-0990).

### Prototype for Individual Prognosis of Depressive Symptoms

The prototype was based on the same data and method as used in the simulation. However, in the implementation of the prototype, time was measured in days, not in fixed time categories; therefore, all measurements were available. The data used and displayed graphically represent concrete individuals and should not, even theoretically, be identifiable. Before transfer to the prototype server, all data were anonymized and identification numbers replaced with random numbers.

## Results

Characteristics of the population are given in Table 1. Male patients constituted 63.60% (893/1404) of the population; there were only minor differences in the distribution of characteristics between genders. The mean HADS-D score in the first measurement was 4.48 (SD 3.94). At that time, 208 (14.81%) of the patients had possible signs of depression (score >7-10) and 116 (8.26%) had definite signs of depression (score >10). The 1404 patients contributed with a total of 7273 questionnaires. The median number of questionnaires per patient was five (interdecile range [IDR] 4-7 questionnaires).

A total of 7181 questionnaires could be uniquely classified into one of the predefined time categories (Table 2). In 92 cases, more than one questionnaire was received from the same patient within the same time category. The first one was included in the simulation study, whereas in the prototype, where time is treated as a continuous variable, all questionnaires were eligible. Full-length questionnaires constituted 69.28% (4975/7181) of all questionnaires, whereas a brief questionnaire was received in 30.72% (2206/7181).

The population’s distribution of HADS-D scores across time categories is summarized in Table 3. The mean scores varied only slightly over time. The median intraindividual standard deviation was 1.38 (interquartile range [IQR] 0.71-2.12). A detailed analysis of the time trend will be published elsewhere.

**Table 1:** Characteristics of stroke patients included in simulation study and prototype (N=1404).

Variable		Female (n=511) n (%)	Male (n=893) n (%)	*P*^a^
**Age (years)**				.06
	≤60	173 (33.9)	264 (29.6)	
	61-70	164 (32.1)	341 (38.2)	
	71-80	174 (34.1)	288 (32.3)	
**Comorbidity index^b^**				.15
	0	284 (55.6)	510 (57.1)	
	1	74 (14.5)	155 (17.4)	
	2	80 (15.7)	102 (11.4)	
	3	22 (4.3)	31 (3.5)	
	>3	20 (3.9)	29 (3.2)	
	NA	31 (6.1)	66 (7.4)	
**Type of stroke**				.73
	Intracerebral hemorrhage	42 (8.2)	71 (8.0)	
	Ischemic	425 (83.2)	729 (81.6)	
	Unspecified	37 (7.2)	80 (9.0)	
	Missing	7 (1.4)	13 (1.5)	
**Year of stroke**				.97
	2008	39 (7.6)	75 (8.4)	
	2009	158 (30.9)	273 (30.6)	
	2010	171 (33.5)	299 (33.5)	
	2011	143 (28.0)	246 (27.5)	
**Type of hospital**				
	University hospital	199 (38.9)	355 (39.8)	.77
	Regional hospital	312 (61.1)	538 (60.2)	
**Depression score at entry**				
	Normal (<7)	374 (73.2)	706 (79.1)	.04
	Possible signs of depression (7-10)	89 (17.4)	119 (13.3)	
	Definite signs of depression (>10)	48 (9.4)	68 (7.6)	

^a^Data are compared between groups using chi-square test.

^b^Charlson index [27]. Stroke diagnosis not included in calculation.

**Table 2:** Inclusion and follow-up in stroke patients by time after stroke (N=1404).

		Time after stroke (months)								Total
		3	6	12	18	24	30	36	42	
**Inclusion/follow-up, n**										
	From last month category	NA	1033	1307	1395	1344	1276	1032	586	
	Plus entry	1033	274	88	9	0	0	0	0	1404
	Minus exit: dead	0	0	0	3	8	4	12	26	53
	Minus exit: study termination	0	0	0	8	10	120	132	83	353
	Minus exit: attrition	0	0	0	49	50	120	302	231	752
	Minus nonresponse this round	0	114	225	229	244	222	4	0	1038
	Total received questionnaires	1033	1193	1170	1115	1032	810	582	246	7181
**Questionnaire type, n**										
	Full length	102	827	852	812	823	794	541	224	4975
	Brief^a^	931	366	318	303	209	16	41	22	2206
**Data collection method, n (%)**										
	Paper	1031 (99.9)	788 (66.1)	775 (66.2)	735 (65.9)	638 (61.8)	451 (55.7)	398 (68.4)	150 (61.0)	4967 (69.2)
	Web	1 (0.1)	405 (34.0)	395 (33.8)	379 (34.1)	394 (38.2)	359 (44.3)	184 (31.6)	96 (39.0)	2214 (30.8)

^a^HADS score estimated from the MOS Short Form 12-item MH4.

**Table 3:** Hospital Anxiety and Depression Scale depression subscale (HADS-D) scores by time after stroke.

	All	Time after stroke (months)							
		3	6	12	18	24	30	36	42
n	4922	101	818	846	806	820	789	519	223
HADS-D score, mean (SD)	4.5 (3.9)	3.9 (3.6)	4.4 (3.9)	4.7 (3.9)	4.6 (3.8)	4.5 (3.8)	4.4 (3.8)	4.6 (4.2)	4.5 (3.9)
HADS-D score, median (IDR^a^)	4 (0-10)	3 (0-9)	3 (0-10)	4 (0-10)	4 (0-10)	4 (0-10)	3 (0-10)	3 (0-11)	3 (1-10)

^a^IDR: interdecile range.

### Simulation

In the simulation study, 4922 questionnaires were available, corresponding to the number of questionnaires with a valid HADS-D score, whereas an additional 936 brief questionnaires from the same patients where included in the combined approach (Table 4). At 3 months, when most questionnaires were of the brief type, 543 questionnaires were available for the combined method compared to 101 in the other three approaches (Table 4). With the combined method, 5% of the simulations were based on less than 364 donor questionnaires, whereas for the other three approaches, fewer questionnaires were available (n=116, n=86, and n=117, respectively) (Table 4). The mean scores differed only slightly between the approaches, except for the 3-month value, where the combined approach had a mean score of 5.1 compared to 3.9 in the other approaches (difference 1.20, 95% CI 0.43-1.97) (Table 4).

The results of the simulation obtained in the four approaches are presented in Table 5. The mean values of the standard deviation in simulated scores were 40% to 70% higher than those of the true scores with the largest difference in the approach based on genuine HADS scores (Table 5). In all approaches, the variation in simulated scores was largest in the quintiles with the lowest index score. The mean error was close to zero for all approaches. As expected, the mean errors in relation to time were close to zero. The MAEs were consistently highest for the common regression approach and lowest for the combined approach. Compared to the population standard deviation (SD 3.94; Table 3), the MAEs were 0.61, 0.53, 0.57, and 0.58 SD for the four approaches, respectively.

#### Prototype for Individual Prognosis

**Figure 2:**
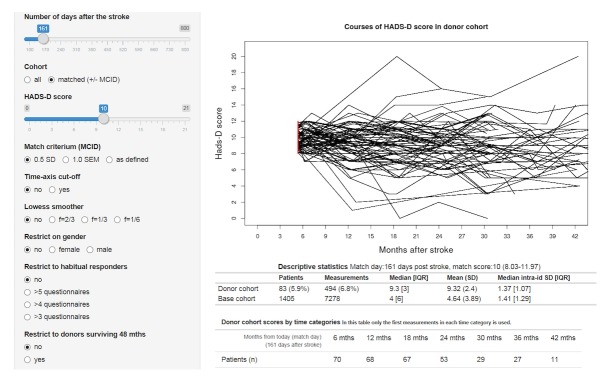
Screenshot (extract) from prototype example: individual prognosis of depressive symptoms for a patient who at 23 weeks poststroke had a HADS-D depression score of 10 points.

In the online prototype, the recipient patient in question is prompted to complete the HADS questionnaire and enter the date of the stroke (Multimedia Appendix 1). Instantly, the courses of depressive symptoms of each member in a subcohort of donors with matching HADS-D score at the same time after the stroke are presented on the screen (Figure 2), together with descriptive statistics of means and variations over time. Furthermore, sentences with suggested wording of prognosis are displayed (Multimedia Appendix 2).

**Table 4:** Numbers of simulated patients and donor patient^a^ questionnaires in a simulation study in a cohort of 1404 patients by four approaches of simulation.

			Genuine HADS-D score	HADS-D estimated from common regression^b^	HADS-D estimated from individual regression^b^	Combined^c^
Included donor questionnaires			Full length questionnaires	Full length questionnaires	Full length questionnaires	Full length questionnaires supplemented with brief questionnaires
**Patients, n**						
	Simulated		1395	1390	1396	1399
	Not simulated^d^		9	14	8	5
**Donor questionnaires, n**						
	Total		1,105,859	1,234,058	1,151,749	1,567,493
	Unique		4922	4922	4922	5858
**Donors and questionnaires per simulation, n**						
	**Minimum**					
		Donor patients	4	4	4	4
		Questionnaires	11	11	11	15
	**5th percentile**					
		Donor patients	29	23	29	63
		Questionnaires	116	86	117	364
	**10th percentile**					
		Donor patients	46	46	46	91
		Questionnaires	186	169	174	516
	**25th percentile**					
		Donor patients	127	127	133	154
		Questionnaires	492	484	493	830
	**Median**					
		Donor patients	199	246	227	211
		Questionnaires	801	919	916	1113
	**Maximum**					
		Donor patients	423	579	423	488
		Questionnaires	1646	2411	1646	2279
**HADS-D score, n; mean (SD)**						
	Overall		4922; 4.5 (3.9)	4922; 4.5 (2.9)	4922; 4.5 (3.8)	5858; 4.6 (3.9)
	3 months		101; 3.9 (3.6)	101; 3.9 (3.6)	101; 3.9 (3.6)	543; 5.1 (3.9)^e^
	6 months		818; 4.4 (3.9)	818; 4.4 (3.9)	818; 4.4 (3.9)	990; 4.6 (3.9)
	12 months		846; 4.7 (3.9)	846; 4.6 (2.9)	846; 4.6 (3.8)	960; 4.6 (3.8)
	18 months		806; 4.6 (3.8)	806; 4.5 (2.7)	806; 4.6 (3.7)	897; 4.7 (3.9)
	24 months		820; 4.5 (3.8)	820; 4.5 (2.5)	820; 4.5 (3.7)	888; 4.6 (3.8)
	30 months		789; 4.4 (3.8)	789; 4.4 (2.4)	789; 4.4 (3.6)	796; 4.4 (3.8)
	36 months		519; 4.6 (4.2)	519; 4.7 (2.7)	519; 4.6 (4.1)	551; 4.6 (4.2)
	42 months		223; 4.5 (3.9)	223; 4.4 (2.4)	223; 4.6 (3.7)	233; 4.6 (3.9)

^a^Subcohorts of patients with a score matching the score at the index date ±0.5 SD.

^b^Regression based on the SF-12 MH4 item.

^c^Genuine HADS-D score with missing scores estimated by individual regression based on the SF-12 MH4 item.

^d^Less than four donors in the matched subcohort.

^e^*P*=.003.

**Table 5:** Results from simulation of trajectories of depressive symptoms in a cohort of 1404 patients by four approaches of simulation.

		Genuine HADS-D score	HADS-D estimated from common regression^a^	HADS-D estimated from individual regression^a^	Combined^b^
**Standard deviation of scores^c^ by quintiles of recipient’s^d^ initial value, mean values of simulated / true value (% difference)**					
	1st quintile	1.7/0.5 (240)	2.0/0.5 (300)	1.5/0.5 (200)	1.7/0.5 (240)
	2nd quintile	2.0/0.7 (190)	2.1/0.7 (200)	1.8/0.7 (160)	2.0/0.8 (150)
	3rd quintile	2.3/1.4 (60)	2.0/1.4 (40)	2.0/1.4 (40)	2.3/1.5 (50)
	4th quintile	2.6/1.6 (60)	2.1/1.6 (30)	2.3/1.6 (40)	2.4/1.7 (40)
	5th. quintile	2.9/1.7 (70)	2.6/1.7 (50)	2.4/1.7 (40)	2.8/1.7 (60)
	Overall	2.4/1.4 (70)	2.1/1.4 (50)	2.0/1.4 (40)	2.3/1.4 (60)
**Mean error simulated-true value**					
	6 months	NA	NA	NA	0.01
	12 months	–0.02	0.00	–0.05	0.08
	18 months	0.02	–0.16	–0.08	0.13
	24 months	–0.02	–0.04	–0.05	0.07
	30 months	–0.01	–0.02	0.01	0.08
	36 months	–0.07	0.08	0.05	–0.08
	42 months	–0.03	0.01	0.08	0.05
	Overall	–0.02	–0.03	–0.03	0.05
**Mean absolute error**					
	6 months	NA	NA	NA	1.7
	12 months	1.9	2.5	1.9	1.8
	18 months	2.1	2.5	2.1	1.8
	24 months	2.0	2.5	2.0	1.8
	30 months	2.1	2.5	2.0	1.8
	36 months	2.4	2.8	2.3	2.0
	42 months	2.4	2.7	2.3	2.0
	Overall	2.1	2.6	2.1	1.8

^a^Regression based on the SF-12 MH4 item.

^b^Genuine HADS-D score with missing scores estimated by individual regression based on the SF-12 MH4 item.

^c^In subcohorts of patients with a score matching the score at the index date ±0.5 SD.

^d^The cohort member for which the prognosis is simulated.

## Discussion

This paper describes a generic method for providing quantitative measures of individual prognosis for PRO-based outcomes. The method is nonparametrical and directly based on original cohort data with repeated PRO assessments.

The mean simulated scores differed only slightly between approaches, except for the 3-month score, where the combined approach had a mean score of 5.1 compared to 3.9 in the other three approaches. Most studies have found that the prevalence of depression is highest in the first period after the stroke [11]. In the first 4 months of data collection, patients were asked to complete the long version of the questionnaire after 3 months, but due to concern about low response rates, we changed the protocol in April 2009 based on the assumption that patients at this early point would more likely complete a briefer version. At 3 months, 931 answered the brief questionnaire, whereas 102 answered the full-length version. The response rate was 69.2% before the change and 81.3% after. Therefore, the lower score in the 101 patients with a genuine HADS-D score at 3 months may be explained by selection bias. The mean 3-month score from the combined approach, which also utilizes data from the brief questionnaires, was more in accordance with other findings, which supports the validity of this approach.

The mean variations in the simulated scores were higher than in the original data (recipients), but did not differ between approaches (Table 5). The variation was, however, only approximately 56% of the overall standard variation (2.2 vs 3.9), which indicates that the method captures additional information. The MAEs were, rather constantly, 2 points, thus in the range of one-half standard deviation among all cohort members. There are two sources for a higher variation in the simulated scores. First, in each simulation, a deviation of up to one-half standard deviation was allowed when selecting donors. Second, the only input used was the actual depression score and the time elapsed since the stroke. In theory, an advanced statistical model would be able to utilize information from more covariates and possibly increase the precision. However, the actual HADS-D score was by far the most important predictor for future HADS scores (data not shown), and other longitudinal studies with similar design have not identified factors of importance for different time trends (ie, interaction on the association between score and time), and the whole model only explained a small percentage of the variation [26]. If previous scores were available for the recipient, not only donor patients with similar actual scores but also those with a similar previous trajectory could be selected. However, given the practical aim of this method, historical scores will typically not be available when the prognosis is requested.

### Strengths and Limitations

The internal validity of the method is high, evaluated as the ability to reproduce values from the original cohort. With respect to external validity, this method shares some limitations with model-based methods. The number of severely depressed patients was low, which may be due to underrepresentation of such patients in the original cohort. In etiologic research, selection bias is potential devastating, and it is likely that patients who comply fully with a protocol differ from patients who only answer a few questions or stop answering completely. However, in the setting of data-based individual prognosis, selection and attrition are dynamic, not static, phenomena because all information regarding future outcomes in the donor cohort is conditioned on surviving (literally and as cohort member) until the index date on which a prognosis is requested. Attrition before that date is therefore merely a question of a reduced number of donors, but given the previously mentioned low predictive values of other covariates, it is less likely to interfere with the distribution of the next measurement in the donor cohort. Nevertheless, the probability of receiving an answer from each donor patient at the next time point will most likely be dependent on the actual health of the patient. Thus, a very good health condition in a patient as well as a very bad one may reduce the probability of answering. This health condition is unknown and unobservable. The combined approach with short questionnaires could be part of a solution, but severely depressed patients will probably still be underrepresented at any point of follow-up. In etiologic research, multiple imputations are often suggested as a solution [28]. However, imputation introduces extra variation, which is a minor problem in etiological research in which the model may only explain a small fraction of the variation, but in the present setting, one of the purposes is to describe the variation itself.

A major strength of the data-based method is its face validity (ie, the intuitive and simple principle easily explainable to clinician and patient), because the prognosis is based on actual scores from actual stroke patients who had reported similar depressive symptoms at the similar point of time after the stroke. This is also the bearing and appealing principle of patient-initiated data capture tools such as PatientsLikeMe [29,30]. However, PRO data collected systematically from a well-defined cohort according to a protocol may be less prone to bias than data collection based on self-selection.

The method is versatile and easy to implement, given that relevant cohort data are available. However, being nonparametric, this method has the disadvantage that subgroups can only be analyzed by stratification, and prediction is only possible for patients who have a combination of covariates that also appear in the source material. However, because only one important predicting variable is involved (the actual score), this is a minor problem, but heterogeneity in the trajectory with respect to a certain covariate cannot be generally ruled out. In the prototype, it is possible to select strata and apply automated in situ nonparametric tests of differences in trajectories between strata of gender, age group, and comorbidity. If other patient cohorts with depression scores are available, it will also be possible to test whether the trajectories in such donor patients differ from those in the actual donor patients and, if not, these patients’ trajectories may be included and provide a merged larger cohort for prognosis for severely depressed patients.

The method described here is for application on the individual level and for descriptive use. In analytic epidemiology, when causal factors are searched for (etiology or prediction), parametric or at least semiparametric methods are needed. In case of repetitive data, group-based trajectory modeling may identify latent strata in the longitudinal data [31]. The output of a group-based trajectory model includes estimated probabilities of group membership for each individual [32]. If data on relevant covariates are present, it may, in theory, be possible to also use such models at the individual level to predict future patterns. It would be highly relevant to compare performance of such models with the present model-free method.

### Parallel Use of Patient-Reported Outcomes for Multiple Purposes

With the increased application of PRO for clinical use, research, and quality improvement, we will need to address in the near future the problem of the use of multiple different questionnaires for the same patient [10]. An important finding of the simulation study is that shorter questionnaires (eg, for clinical use) may not only coexist with longer questionnaires (for research, quality improvement, as well as use for individual prognosis), but may even provide less-biased longitudinal data given that the design is prepared for individual regression by including at least two simultaneous measurements of the full scale and the surrogate measure.

### Access to Prototype

The online prototype of the implementation for depressive symptoms after stroke is available at the website www.prognosis.dk [33].

### Conclusion

Internal simulation in a population of stroke patients showed almost similar results in four different approaches, but an approach in which missing scores were calculated based on individual regression coefficients performed best in terms of validity. This is the first description and implementation of a nonparametric cohort-based method for individual prognosis. Further elaborations will be developed and evaluated, and the usability and clinical validity [34] of the method in clinical practice will be scrutinized.

## Appendix

Multimedia Appendix 1 Landing page for http://prognosis.dk.

Multimedia Appendix 2 Example of individual prognosis of depressive symptoms after stroke for a patient who 23 weeks after the stroke has a HADS-D score of 10.

## Abbreviations

HADS: Hospital Anxiety and Depression Scale
MAE: mean absolute error
MCID: minimal clinically important difference
MOS: Medical Outcomes Study
PRO: patient-reported outcome
