# Supplementary figures and images for: Individual Prognosis of Symptom Burden and Functioning in Chronic Diseases: A Generic Method Based on Patient-Reported Outcome (PRO) Measures
Source: J Med Internet Res. 2017 Aug 1;19(8):e278. doi: 10.2196/jmir.8111 (PMC5558046; doi:10.2196/jmir.8111)

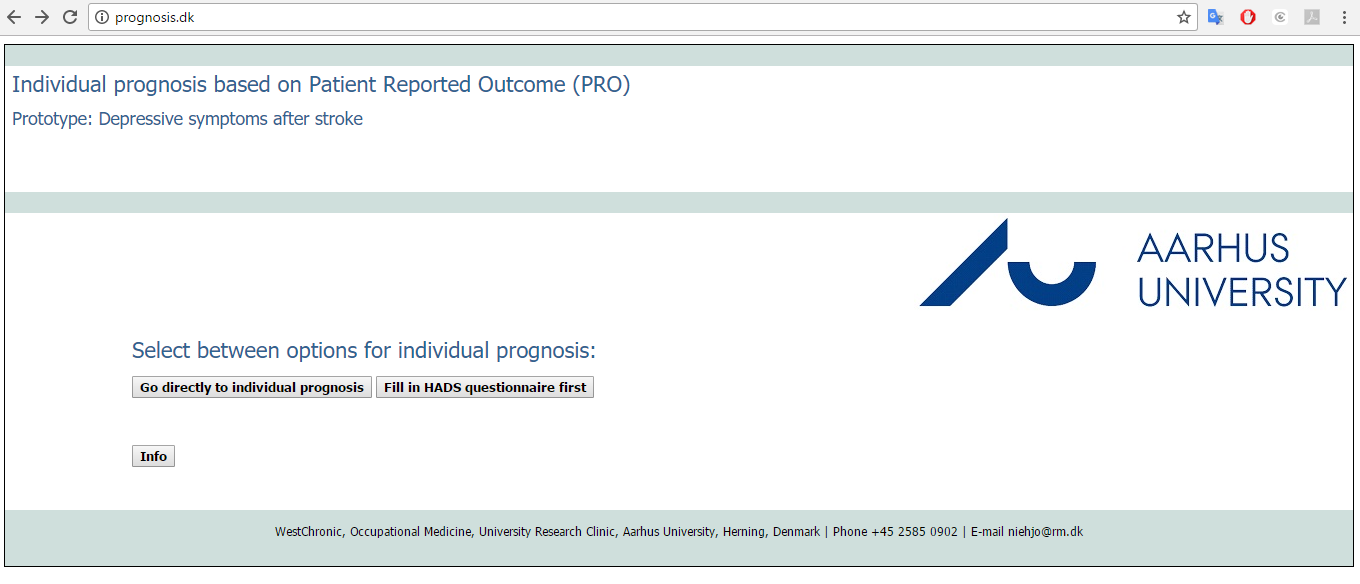

Supplement: Multimedia Appendix 1 [file jmir_v19i8e278_app1.PNG]
