# Supplementary material for: Individual Prognosis of Symptom Burden and Functioning in Chronic Diseases: A Generic Method Based on Patient-Reported Outcome (PRO) Measures
Source: J Med Internet Res. 2017 Aug 1;19(8):e278. doi: 10.2196/jmir.8111 (PMC5558046; doi:10.2196/jmir.8111)
Supplement: Multimedia Appendix 2 [file jmir_v19i8e278_app2.pdf]

Number of days after the stroke

100 161 800

100 170 240 310 380 450 520 590 660 730 800

Cohort

○ all ◉ matched (+/- MCID)

HADS-D score

0 10 21

0 3 6 9 12 15 18 21

Match criterium (MCID)

◉ 0.5 SD ○ 1.0 SEM ○ as defined

Time-axis cut-off

◉ no ○ yes

Lowess smoother

◉ no ○ f=2/3 ○ f=1/3 ○ f=1/6

Restrict on gender

◉ no ○ female ○ male

Restrict to habitual responders

◉ no

○ >5 questionnaires

○ >4 questionnaires

○ >3 questionnaires

Restrict to donors surviving 48 mths

◉ no

○ yes

Options below are experimental:

Prototype version 1.0, May 2017. Info/FAQ here. (http://prognosis.dk/info3.pdf) Landing page here (http://prognosis.dk)

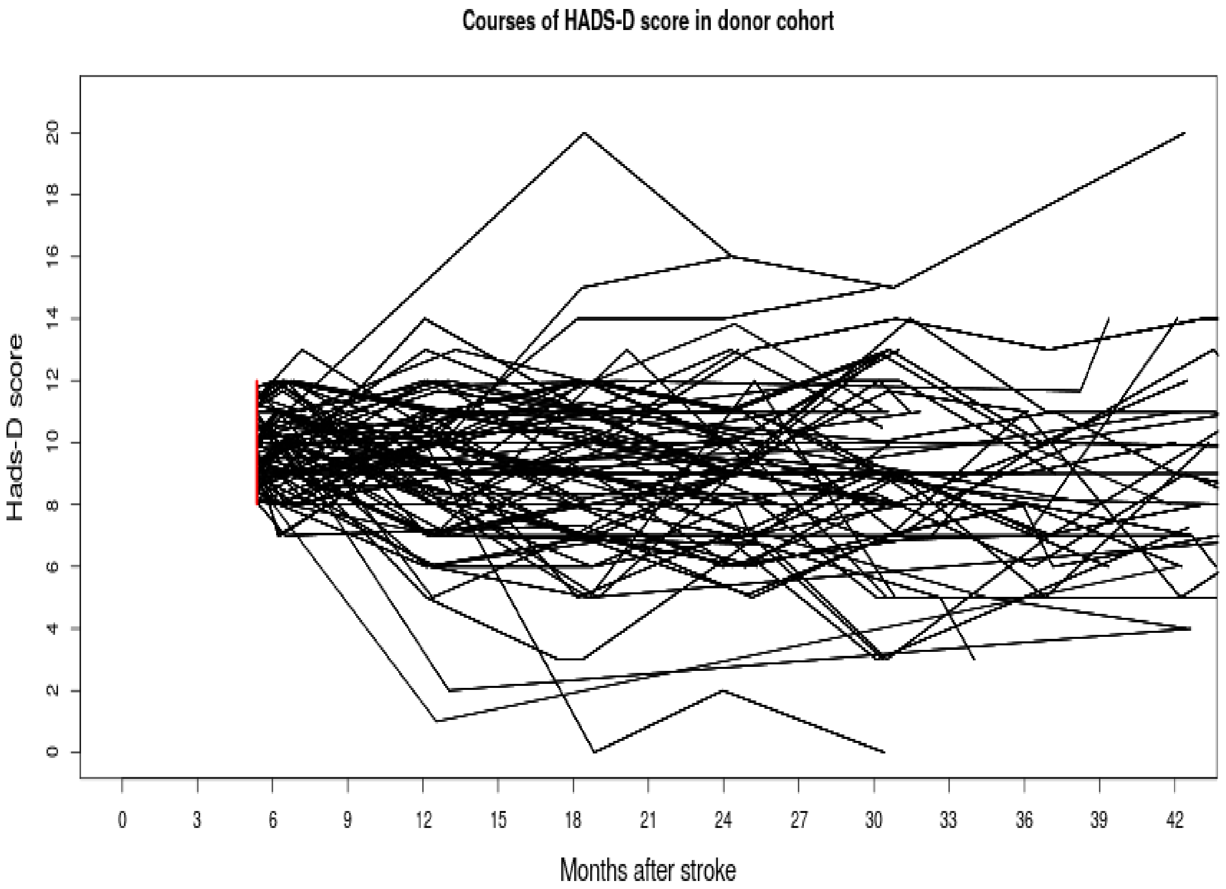

Descriptive statistics Match day:161 days post stroke, match score:10 (8.03-11.97)

	Patients	Measurements	Median [IQR]	Mean (SD)	Median intra-id SD [IQR]
Donor cohort	83 (5.9%)	494 (6.8%)	9.3 [3]	9.32 (2.4)	1.37 [1.07]
Base cohort	1405	7278	4 [6]	4.64 (3.89)	1.41 [1.29]

Donor cohort scores by time categories **In this table only the first measurements in each time category is used.**

Months from today (match day) (161 days after stroke)	6 mths	12 mths	18 mths	24 mths	30 mths	36 mths	42 mths
Patients (n)	70	68	67	53	29	27	11

Suppress plot of unstable donors

- ( ) no
- ( ) >16
- (•) >8

Restricted dataset (Table 2)

- (•) no
- ( ) yes

Use regression estimates from restricted dataset

- (•) no
- ( ) yes

HADS-D scoreOutcome variable

- ( ) genuine HADS-D scores only
- (•) genuine HADS-D scores supplemented with intra-id regression

Use fixed time intervals

- (•) no
- ( ) yes

Index day score by interpolation

- ( ) no
- (•) yes

Donor cohort scores by time categories **In this table only the first measurements in each time category is used.**

Difference (ref:match day score)							
- Improved (%)	27.1	26.5	28.4	30.2	34.5	44.4	27.3
- Stable (%)	62.9	63.2	56.7	56.6	58.6	33.3	63.6
- Worse (%)	10	10.3	14.9	13.2	6.9	22.2	9.1
Difference (ref:preceding score)							
- Improved (%)	27.1	19.6	23.2	27.7	38.1	27.8	33.3
- Stable (%)	62.9	60.7	57.1	42.6	38.1	38.9	50
- Worse (%)	10	19.6	19.6	29.8	23.8	33.3	16.7
Cummulative experiences							
- No change (%)	62.9	51.5	32.8	22.6	20.7	7.4	9.1
- Changes (%)	37.1	48.5	67.2	77.4	79.3	92.6	90.9
- - Improvements (%)	27.1	32.4	43.3	52.8	58.6	66.7	36.4
- - Worsenings (%)	10	17.6	26.9	28.3	20.7	29.6	54.5

Individualized prognostic statements:

The prognosis for your depressive symptoms is based on how previous patients fared who at the same time after their stroke as you (5.4 months) had similar depressive symptoms (HADS-D score: 10).

Six months later (11.4 months after the stroke) 10 % of the patients had worse depressive symptoms,27.1 % had fewer symptoms while 62.9 % had symptoms like you have now. One year later (17.4 months after the stroke) 10.3 % of the patients had worse depressive symptoms, 26.5 % had fewer symptoms while 63.2 % had symptoms like you have now.

Notice that the courses of depressive symptoms are very variable. Over a 2-year period, 77.4 % of patients like you reported changed depressive symptoms (better or worse) in at least one questionnaire and over the course of 3 years it was 92.6 %

Like the weather forecast, the prognosis becomes more uncertain the longer we look forward, for example several years. You can at anytime revisit this page to obtain a new and more reliable short-term prognosis.
